# What is the main factor in predicting the morbidity and mortality in patients with gastroschisis: delivery time, delivery mode, closure method, or the type of gastroschisis (simple or complex)?

**DOI:** 10.3906/sag-2011-166

**Published:** 2021-06-28

**Authors:** Mustafa BEHRAM, Süleyman Cemil OĞLAK*, Seyithan ÖZAYDIN, Sema Süzen ÇAYPINAR, İlker GÖNEN, Şeyhmus TUNÇ, Yusuf BAŞKIRAN, İsmail ÖZDEMİR

**Affiliations:** 1 Department of Perinatology, Health Sciences University, Kanuni Sultan Süleyman Training and Research Hospital, İstanbul Turkey; 2 Department of Obstetrics and Gynecology, Health Sciences University, Gazi Yaşargil Training and Research Hospital, Diyarbakır Turkey; 3 Department of Pediatric Surgery, Health Sciences University, Kanuni Sultan Süleyman Training and Research Hospital, İstanbul Turkey; 4 Department of Neonatology, Health Sciences University, Kanuni Sultan Süleyman Training and Research Hospital, İstanbul Turkey

**Keywords:** Gastroschisis, complex gastroschisis, enteral feeding, wound infection, mechanical ventilation, abdominal wall closure method

## Abstract

**Background/aim:**

There are numerous debates in the management of gastroschisis (GS). The current study aimed to evaluate perinatal outcomes and surgical and clinical characteristics among GS patients based on their type of GS, abdominal wall closure method, and delivery timing.

**Materials and methods:**

This study was a retrospective analysis of prospectively collected data of 29 fetuses with GS that were prenatally diagnosed, delivered, and managed between June 2015 and December 2019 at the Obstetrics and Pediatric Surgery Clinics of Kanuni Sultan Süleyman Training and Research Hospital.

**Results:**

Twenty-three of the patients had simple GS, and six of them had complex GS. The reoperation requirement, number of operations, duration of mechanical ventilation, time to initiate feeding, time to full enteral feeding, total parenteral nutrition (TPN) duration, TPN-associated cholestasis, wound infection, sepsis, and necrotizing enterocolitis were significantly lower in the simple GS group than in the complex GS group. The mean hospital length of stay was 3.5 times longer in the complex GS group (121.50 ± 24.42 days) than in the simple GS group (33.91 ± 4.13 days, p = 0.009). There were no cases of death in the simple GS group. However, two deaths occurred in the complex GS group.

**Conclusion:**

This study indicated that simple GS, compared with complex GS, was associated with improved neonatal outcomes. We suggest that the main factor affecting the patients’ outcomes is whether the patient is a simple or complex GS rather than the abdominal wall closure method.

## 1. Introduction

Gastroschisis (GS) is a congenital malformation of the anterior abdominal wall, commonly to the right of a normally inserted umbilical cord and less than 4 cm in diameter. This defect results in the evisceration of the fetal intestines and occasionally other intraabdominal organs outside the abdomen that is not covered by a sac or membrane [1]. GS occurs in 3–4.5 in approximately 10,000 live births with a male predominance [2]. The prevalence of GS demonstrates an uptrend worldwide with a strong association with young (<20 years) maternal age [3]. Although the real pathogenesis remains unclear, GS likely results from the rupture of the physiological hernia [4]. Possible factors have been cited for this defect, including vasoconstrictive agents, smoking, illicit drugs, acetylsalicylic acid, oral contraceptives, and environmental teratogens. Patients with GS do not typically have associated chromosomal anomalies but are more likely to have structural gastrointestinal abnormalities in 10% of the cases [5]. Many of them are born preterm and are often small for gestational age (SGA) [6].

GS is typically diagnosed prenatally using fetal ultrasonography (US) with a specificity of >95% [5]. It is commonly viewed in midsecond trimester fetal US with features of a right-sided defect with free-floating intestine in the amniotic cavity. Increased α-fetoprotein levels in both amniotic fluid and maternal blood have been linked with this defect [6]. Following the prenatal diagnosis of GS, parental counseling about the fetus’s treatment and prognosis, and referral to the patient to a tertiary center with maternal-fetal medicine, genetic counseling, neonatology, and pediatric surgery is suggested [7]. Thus, those patients maintain a close follow-up to predict and prevent adverse outcomes associated with GS, including premature delivery, oligohydramnios, SGA, and fetal death [5]. Also, neonates with GS may endure prolonged hospital length of stay (LOS) due to prematurity complications, bowel inflammation, ischemia or atresia, general anesthesia exposure, wound infection, sepsis, prolonged ventilator support, impaired intestinal function, and necessity of total parenteral nutrition (TPN) [8]. In 17% of GS patients, the exposed bowel is vulnerable to injuries due to additional defects, including intestinal atresia, necrosis, perforation, or volvulus, labeled as complex GS. Those patients have a higher risk of morbidity and mortality than neonates without additional intestinal defects, labeled as simple GS [9].

The morbidity of GS is principally determined by the severity of the bowel damage existing at birth, and postnatal management purposes include reducing the bowel back into the abdominal cavity without trauma to the intestine, closure of the abdominal wall defect, avoiding increased intraabdominal pressure and enteral feed initiation [10]. However, there are numerous debates in the management of GS, including mode of delivery, delivery timing, and the abdominal wall closure method. A metaanalysis suggested elective preterm delivery to reduce intrauterine intestinal exposure to the amniotic fluid’s toxic environment, to prevent demise and ameliorate intestinal injury, while another metaanalysis stated the association between earlier gestational age at delivery and adverse neonatal outcomes [11,12]. Similarly, some researchers have advocated performing routine cesarean delivery to avoid bowel injury, and cesarean rates among neonates with GS were high. Still, current literature has demonstrated that outcomes were not influenced by delivery mode [13]. Additionally, the optimal abdominal wall closure method is still debated among the clinicians. Following the development of the spring-loaded preformed silo, the staged closure method has been used more often, with several studies demonstrating comparable results to the primary closure method [6,8]. 

This study sought to evaluate perinatal outcomes, surgical and clinical characteristics among GS patients based on their type of GS, abdominal wall closure method, and delivery timing at our tertiary center, where early-term delivery is routinely practiced.

## 2. Methods

### 2.1. Study design

The current study was a retrospective analysis of prospectively collected data of fetuses with GS that were prenatally diagnosed, delivered, and managed between June 2015 and December 2019 at the Obstetrics and Pediatric Surgery Clinics of Training and Research Hospital. All pregnant women were examined by a maternal-fetal medicine (MFM) specialist with the GE Voluson E6 (General Electric Healthcare, Milwaukee, WI, USA) device, and the perinatology council decided the definitive diagnosis. We excluded neonates with genetic syndromes, chromosomal abnormalities, congenital infectious diseases, and stillbirths. We also excluded neonates with incomplete medical records, follow-up losses, and parents unwilling to participate in this study. The Ethics Committee of Kanuni Sultan Süleyman Training and Research Hospital approved the study (2020.07.160). We obtained informed consent forms from all parents.

A total of 29 patients enrolled in the study. Twenty-three of them had simple GS, and six of them had complex GS. We defined complex gastroschisis as the presence of intestinal atresia, perforation, and necrosis at birth, or those who developed short gut syndrome (SGS) and became dependent on TPN for more than 60 days (Molik’s definition) [14]. 

We recorded demographic and baseline data and surgical and clinical characteristics of the patients. The demographic and baseline characteristics were as follows: maternal age, parity, smoking, delivery mode, gestational age at birth, the presence of preterm birth and SGA, birth weight, and Apgar scores at 1 and 5 min after birth. Early-preterm birth was defined as delivery before 35 weeks of gestation. Late-preterm birth was defined as delivery between 350/7 and 366/7 weeks of gestation. We defined early-term birth as delivery between 370/7 and 386/7 weeks of gestation [8]. SGA was defined as a birth weight less than the 10th percentile for gestational age [15]. In our clinic, early-term delivery is routinely practiced, unless maternal and fetal reasons require otherwise. The surgical characteristics were as follows: silo requirement, duration with a silo, and requirement for reoperation.

According to our clinical protocol, a pediatrician and a pediatric surgeon are present in the delivery room during the birth of all neonates with GS. Following the birth of the infant, fluid resuscitation and gastric decompression are immediately initiated. Since these patients experience significant evaporative and heat loss due to the exposed viscera, the bowel is wrapped in warm, saline-soaked gauze, and the lower half of the infant is placed in a bowel bag. All patients underwent surgery under general anesthesia within 6 h after delivery. A nasogastric tube was inserted into the stomach and a catheter into the bladder to decompress the intestine distension and provide more space inside the abdomen. According to Molik’s definition, eviscerated organs were evaluated and determined whether the patient had simple or complex GS. The primary reduction was preferred if the surgeon was able to securely place the eviscerated organs into the abdominal cavity without causing excessive intraabdominal pressure and vital instability. If the primary reduction was not feasible, the eviscerated content was placed into a transparent, spring-loaded, preformed silo. The ring at the base of the silo is placed into the abdomen through the defect. Then the surgeon performed serial reductions daily or twice a day over 7 to 10 days with the help of gravity until the contents reached the fascia level. This staged reduction allows the gradual reduction of bowel edema and allows for bowel reduction without increasing intraabdominal pressure. Primary suture technique was used as abdominal wall closure method in all patients. The fascia was closed; then, the skin was sutured while protecting the umbilicus. Also, if the cause of complex GS was intestinal atresia, the defect was closed, and a second surgery was performed within a few weeks to constitute bowel continuity. 

The clinical characteristics were as follows: need for mechanical ventilation, duration of mechanical ventilation, time to initiate feeding, time to reach full enteral feeding, duration of TPN, TPN-associated cholestasis, surgical site infection, sepsis, necrotizing enterocolitis (NEC), abdominal compartment syndrome, hospital length of stay (LOS), and patients discharged as death. We defined time to full enteral feeding as achieving 150 mg/kg/day. We defined TPN-associated cholestasis as direct bilirubin greater than 2 mg/dL. We identified surgical site infection based on wound erythema, purulent discharge or pus, and antibiotics treatment. We defined sepsis as blood culture-proven patients only. NEC was defined as either surgically identified pneumatosis intestinalis or portal venous gas on imaging.

Since the visceral exposure increases infection risk, we initiated antibiotics for all patients after birth and discontinued them after 10–14 days if the patient was clinically stable without infection signs. If there is a silo in place, antibiotics were continued until its removal. In case of sepsis, antibiotics were chosen according to the blood culture result. TPN was initiated in all patients on the first day of life as certain amount of time would be required to initiate enteral feeding. We initiated formula or exclusive breast milk to the patient after gaining the intestinal adaption and motility. Since there is no home TPN program in our country yet, patients were hospitalized during the TPN therapy. Patients who were fed full enterally were discharged in the presence of normal infection parameters.

### 2.2. Statistical analysis

Data were summarized as mean ± standard error of the mean (SEM) for continuous variables, frequencies (percentiles) for categorical variables. For comparisons, nonparametric test methods were used due to the small number of subjects per group. The Mann–Whitney U test was used for two independent groups and the Kruskal–Wallis for more than two independent group comparisons. Chi-square test was used for proportions, and its counterpart Fisher’s exact test was used when the data were sparse. When the p-value from the Kruskal–Wallis test statistics is statistically significant, pairwise comparisons were used to know which time point differ from which others. All statistical analyses were performed by using IBM SPSS 20.0 for Windows (IBM Corp., Armonk, NY, USA). A p-value less than 0.05 was considered statistically significant. 

## 3. Results

During the study period, 37 cases with GS were prenatally diagnosed and followed up. Of these, two pregnancies were terminated due to additional major congenital abnormalities. Three fetuses died in utero at 18, 31, and 33 weeks of gestation. The fetus that died at 18 weeks of gestation had additional major congenital abnormalities. The other two of the intrauterine fetal demise cases showed intrauterine growth restriction and bowel dilatation. Three patients were delivered and treated in other hospitals, and parents refused to participate in the study. 

A total of 29 patients were included in the study. Of these infants, 23 (79.3%) were diagnosed with simple GS, and 6 (20.7%) were diagnosed with complex GS. Of the patients with complex GS, 4 had intestinal atresia, 2 had intestinal atresia and perforation, and 1 had intestinal perforation only. Also, of all patients with GS, 8 patients had stomach evisceration, and 4 patients had bladder evisceration.

We presented the demographic and baseline characteristics of the study cohort and the comparison between simple and complex GS groups in Table 1. There were no significant differences between the two groups in terms of maternal age, smoking, fetal sex, birth week, prematurity, cesarean section, SGA presence, and Apgar scores at 1 and 5 min after birth. Birth weight was significantly lower in the complex GS group (1857.50 ± 140.58 g) than the simple GS group (2374.34 ± 129.14, p = 0.016). 

**Table 1:** Demographic and baseline characteristics of the participants.

	All patients (n = 29)	Simple GS (n = 23)	Complex GS (n = 6)	p-value
Maternal age, years	21.2 ± 0.68	21.7 ± 0.54	20.9 ± 1.67	0.290
Smoking, n (%)	4 (13.8%)	3 (13.04%)	1 (16.6%)	0.471
Sex, n (%)				0.651*
Male	14 (48.0%)	12 (52.2%)	2 (33.3%)	
Female	15 (52.0%)	11 (47.8%)	4 (66.7%)	
Birth week	33.93 ± 0.45	34.08 ± 0.54	33.33 ± 0.84	0.517
Prematurity, n (%)	23 (79.3%)	17 (73.9%)	6 (100%)	0.295*
Delivery time, n (%)				0.406*
Early-preterm	17 (59.0%)	13 (56.5%)	4 (66.7%)	
Late-preterm	6 (20.5%)	4 (17.4%)	2 (33.3%)	
Early-term	6 (20.5%)	6 (26.1%)	0 (0%)	
Cesarean section, n (%)	25 (92.5%)	19 (82.6%)	6 (100%)	0.553*
Birth weight, g	2267.41 ± 112.64	2374.34 ± 129.14	1857.50 ± 140.58	0.016
SGA, n (%)	15 (48.2%)	11 (47.8%)	3 (50.0%)	0.337
1-min Apgar	7.4 ± 0.11	7.6 ± 0.12	7.3 ± 0.16	0.665*
5-min Apgar	8.2 ± 0.09	8.5 ± 0.12	8.1 ± 0.20	0.883*

Note: The values are presented as mean ± SEM and n (%). * Fisher’s exact p-value for categorical variables. GS: gastroschisis, TPN: total parenteral nutrition, SGA: small for gestational age.

We demonstrated the surgical and clinical characteristics of the simple and complex GS patients in Table 2. Staged abdominal wall closure with silo rates and duration of the silo were similar between the two groups. All patients experienced mechanical ventilation. The reoperation requirement, number of operations, duration of mechanical ventilation, time to initiate feeding, time to full enteral feeding, TPN duration, TPN-associated cholestasis, wound infection, sepsis, and NEC were significantly higher in the complex GS group than the simple GS group. All patients required reoperation in the complex GS group, while 47.8% of simple GS patients required reoperation. The indications for reoperation in all GS patients were intestinal atresia, adhesive bowel obstruction, and NEC. The mean hospital LOS was 3.5 times longer in the complex GS group (121.50 ± 24.42 days) than that of the simple GS group (33.91 ± 4.13 days, p = 0.009). There were no cases of death in the simple GS group. However, two deaths occurred in the complex GS group. They were both born at 33 weeks of gestation. Both of them died at the age of 6 months. One of them died because of short bowel syndrome, and the other died of sepsis. 

**Table 2:** Surgical and clinical characteristics of the patients.

	All patients (n = 29)	Simple GS (n = 23)	Complex GS (n = 6)	p-value
Silo, n (%)	7 (24.0%)	5 (21.7%)	2 (33.3%)	0.612*
Duration of silo, days	9.28 ± 0.31	8.60 ± 0.27	11.00 ± 0.57	0.190
Reoperation, n (%)	17 (59.0%)	11 (47.8%)	6 (100%)	0.028*
Number of operations	2.24 ± 0.24	1.78 ± 0.19	4.00 ± 0.44	0.005
Mechanical ventilation, n (%)	29 (100%)	22 (100%)	7 (100%)	N/A
Duration of mechanical ventilation, days	10.31 ± 1.93	6.00 ± 1.13	26.83 ± 3.30	<0.001
Time to initiate feeding, days	24.31 ± 2.91	18.91 ± 2.62	45.00 ± 2.58	<0.001
Full enteral feeding, days	45.37 ± 5.26	26.73 ± 3.41	114.25 ± 18.19	<0.001
Duration of TPN, days	42.44 ± 8.85	24.17 ± 3.24	112.50 ± 26.63	<0.001
TPN-associated cholestasis, n (%)	11 (37.9%)	5 (21.7%)	6 (100%)	0.001*
Wound infection, n (%)	7 (25.0%)	1 (4.5%)	6 (100%)	<0.001*
Sepsis, n (%)	10 (34.5%)	4 (17.4%)	6 (100%)	<0.001*
NEC, n (%)	6 (20.6%)	2 (8.6%)	4 (66.6%)	0.002*
Hospital LOS, days	52.03 ± 8.81	33.91 ± 4.13	121.50 ± 24.42	0.009
Death, n (%)	2 (6.8%)	0 (0%)	2 (33.3%)	0.431*

Note: The values are presented as mean ± SEM and n (%). * Fisher’s exact p-value for categorical variables. GS: gastroschisis, TPN: total parenteral nutrition, NEC: necrotizing enterocolitis, LOS: length of stay.

We performed a separate analysis to compare neonatal outcomes by the closure type (Table 3). There were no significant differences between the primary closure and delayed closure with silo in terms of reoperation requirement, the number of operations, duration of mechanical ventilation, time to initiate feeding, time to full enteral feeding, duration of TPN, TPN-associated cholestasis, wound infection, sepsis, NEC, and hospital LOS. 

**Table 3:** Surgical and clinical outcomes for patients with GS by closure type analysis.

	All patients (n = 29)	Primary closure (n = 22)	Silo (n = 7)	p-value
Prematurity, n (%)	23 (79.3%)	18 (81.8%)	5 (71.4%)	0.612*
Birth week	33.93 ± 0.45	34.00 ± 0.48	33.71 ± 1.26	0.567
Birth weight, g	2267.41 ± 112.64	2296.59 ± 134.01	2175.71 ± 207.65	0.145
Complex GS	6 (16.0%)	4 (18.2%)	2 (28.6%)	0.612
Duration of silo, days	9.28 ± 0.31	-	9.28 ± 0.64	N/A
Reoperation, n (%)	17 (59.0%)	11 (50.0%)	6 (85.7%)	0.187*
Number of operations	2.24 ± 0.24	2.13 ± 0.30	2.57 ± 0.36	0.256
Duration of mechanical ventilation, days	10.31 ± 1.93	10.27 ± 2.34	10.42 ± 3.43	0.798
Time to initiate feeding, days	24.31 ± 2.91	23.18 ± 3.36	27.85 ± 6.09	0.518
Full enteral feeding, days	45.37 ± 5.26	44.20 ± 4.11	46.28 ± 17.23	0.276
Duration of TPN, days	42.44 ± 8.85	41.90 ± 10.58	44.14 ± 16.04	0.665
TPN-associated cholestasis, n (%)	11 (38.0%)	9 (40.9%)	2 (28.6%)	0.677*
Wound infection, n (%)	7 (25.0%)	5 (23.8%)	2 (28.6%)	0.801
Sepsis, n (%)	10 (34.5%)	7 (31.8%)	3 (42.9%)	0.665*
NEC, n (%)	6 (20.6%)	4 (17.3%)	2 (28.6%)	0.538*
Hospital LOS, days	52.03 ± 8.81	51.72 ± 10.33	53.00 ± 18.47	0.680
Death, n (%)	2 (6.8%)	2 (9.1%)	0 (0%)	1.000 *

Note: The values are presented as mean ± SEM and n (%). * Fisher’s exact p-value for categorical variables. GS: gastroschisis, TPN: total parenteral nutrition, NEC: necrotizing enterocolitis, LOS: length of stay.

When we divided the patients into three groups according to their gestational age at delivery (early-preterm, late-preterm, and early-term) and compared them, the groups were similar in terms of reoperation requirement, the number of operations, duration of mechanical ventilation, time to initiate feeding, time to full enteral feeding, duration of TPN, TPN-associated cholestasis, wound infection, sepsis, NEC, and hospital LOS (Table 4). There was no complex GS patient in the early-term group.

**Table 4:** Surgical and clinical outcomes for patients with GS by gestational age at delivery.

	Early-preterm (n = 17)	Late-preterm (n = 23)	Early-term (n = 6)	p-value
Birth week	32.29 ± 0.44a	35.50 ± 0.11b	37.44 ± 0.09b	<0.001
Birth weight, g	1985.58 ± 127.46a	2436.66 ± 59.70ab	2896.66 ± 229.26a	0.002
Complex GS	4 (23.5%)	2 (33.3%)	0 (0%)	0.406*
Silo, n (%)	4 (23.5%)	1 (16.7%)	2 (33.3%)	0.857*
Duration of silo, days	9.25 ± 0.36	12.00 ± 0.10	8.00 ± 0.20	0.654
Reoperation, n (%)	10 (58.8%)	4 (66.7%)	3 (50.0%)	1.000*
Number of operations	2.35 ± 1.41	2.66 ± 1.50	1.50 ± 0.54	0.280
Duration of mechanical ventilation, days	12.00 ± 2.85	10.50 ± 2.15	5.33 ± 1.80	0.591
Time to initiate feeding, days	27.05 ± 3.78	27.00 ± 3.73	13.83 ± 4.43	0.197
Full enteral feeding, days	51.06 ± 4.28	51.83 ± 10.31	20.16 ± 6.36	0.148
Duration of TPN, days	48.35 ± 13.07	49.66 ± 10.07	18.50 ± 6.44	0.096
TPN-associated cholestasis, n (%)	7 (41.2%)	3 (50.0%)	1 (16.7%)	0.587*
Wound infection, n (%)	4 (23.5%)	3 (50.0%)	0 (0%)	0.183*
Sepsis, n (%)	5 (29.4%)	3 (50.0%)	2 (33.3%)	0.861*
NEC, n (%)	3 (17.6%)	2 (8.7%)	1 (16.6%)	0.653*
Hospital LOS, days	58.47 ± 12.59	58.33 ± 10.75	27.50 ± 8.22	0.120
Death, n (%)	2 (11.8%)	0 (0%)	0 (0%)	1.000 *

Note: The values are presented as mean ± SEM and n (%). * Fisher’s exact p-value for categorical variables. ab; Means represented by the same superscript are the same, while the means represented by different superscripts are statistically different. GS: gastroschisis, TPN: total parenteral nutrition, NEC: necrotizing enterocolitis, LOS: length of stay.

## 4. Discussion

In the current study, we assessed perinatal characteristics, surgical and clinical outcomes of patients born with GS based on their type of GS, abdominal wall closure method, and delivery timing at our tertiary center. We found that simple GS, compared with complex GS, was associated with improved neonatal outcomes, including reoperation requirement, the number of operations, duration of mechanical ventilation, time to initiate feeding, time to full enteral feeding, duration of TPN, TPN-associated cholestasis, wound infection, sepsis, NEC, and hospital LOS. However, we did not find an association between the closure type of GS and neonatal outcomes. Also, neonatal outcomes of infants born during early-preterm, late-preterm, and term periods were similar. 

The optimal delivery mode for fetuses with GS is controversial. The current literature does not advocate routine cesarean delivery for GS patients, and it is recommended to determine the delivery mode based on obstetrical indications [14]. Slater and Pimpalwar stated that the delivery mode should be at the discretion of the clinician and parents [6]. Palatnik et al. said that they did not routinely perform a cesarean section in patients with GS, and they did not control the delivery time of these infants. They reported that neonates with GS delivered during night time received delayed closure more frequently [8]. We planned delivery during daytime hours to reach relevant specialists. Therefore, 92.5% of our patients underwent a cesarean section.

In our study cohort, 59.0% of the patients were early-preterm, and 20.5% were late-preterm. Most of our patients with GS were categorized in the simple GS group (79.3%). Infants with complex GS comprised the 20.7% of all patients. This frequency was slightly higher than the previous report’s frequency of 17% [9]. Complex GS, defined by the presence of additional intestinal defects such as ischemia, perforation, stenosis, or atresia is associated with increased morbidity and mortality [1]. Consistent with the literature, in our study, complex GS patients had poorer perinatal, surgical, and clinical outcomes than simple GS patients. Also, two infants with complex GS died without reaching full enteral feeding, while there were no death cases in the simple GS group. This difference was not statistically significant. We think that the absence of the statistical difference is due to the low sample size.

Neonates with GS are at a greater risk of nosocomial and opportunistic infections because of their comorbidities and immunological status. These patients often require additional surgical interventions, prolonged time to achieve full enteral feeding, and have extended hospital LOS [16]. Infectious complications (ICs) such as wound infections and sepsis have been demonstrated to affect outcomes, including hospital LOS and mortality [17]. Uribe-Leitz et al. reported that two-thirds of all GS patients had ICs, and hospital LOS in patients with ICs was significantly longer in patients without infection [16]. They found that 65% of simple GS patients and 73% of complex GS patients had ICs. Our study results showed a high incidence of ICs in the complex GS group (100%). We considered that our high incidence of ICs in the complex GS group was because of the high frequency (100%) and the number of operations (4.00 ± 0.44) in this group. Woldemicael et al. concluded that GS closure had a higher incidence of surgical site infections than the other laparotomy procedures (54% and 9%, respectively) [18]. The hospital LOS in the simple GS group was similar to the literature (33.91 ± 4.13 days). However, due to our high ICs rates, hospital LOS of the complex GS group was longer than those observed by other studies [14,19,20].

After surgical intervention, patients frequently suffer delayed bowel dysfunction due to intestinal dysmotility. TPN and gastric decompression should be provided during the abnormal intestinal motility period until enteral feeding is initiated [21]. Our time to initiate feeding, time to full enteral feeding, and duration of TPN were similar to those of other recent studies [14,20,22]. Prolonged exposure to TPN and delay in enteral feeding contribute to TPN-associated cholestasis via intestinal villous atrophy, increased mucosal permeability, and bacterial translocation [23]. Fallon et al. reported that 28% of patients with GS developed cholestasis [24]. We observed a higher (37.9%) prevalence of cholestasis. This was due to the higher cholestasis prevalence of our complex GS patients (100%). 

NEC is a severe complication after GS surgery, tends to occur later in the clinical course, is frequently recurrent, and can be responsible for morbidity and occasional mortality. However, NEC can often be successfully treated without surgical intervention [25]. Gupta et al. reported that NEC occurred in 20% of GS patients [26]. In our study, the incidence of NEC in GS patients was 20.6%. 

The abdominal wall closure method is still debated in the clinical practice. Reduction of the intestines to the abdominal cavity depends on the bowel state (ischemia, edema, necrosis, matting) and the abdominal cavity’s adequacy to accommodate the viscera [10]. Also, situational factors (nighttime admission, outborn) and the institution’s clinical practice affect the choice of wall closure method [27]. In 1998, Bianchi et al. suggested elective delayed midgut reduction without anesthesia as a safe technique, carrying no additional morbidity and mortality [28]. We prefer primary closure as the first option for patients who do not have significant edema, distension, or matting of intestinal loops and have an adequate abdominal domain to accommodate the bowel without causing excessive intraabdominal pressure. We performed this technique immediately after birth in the delivery room, which appears to be a similar but safer and more feasible method than the traditional Bianchi procedure. Patients with distended or very thickened bowel and inadequate abdominal capacity underwent a staged reduction of the intestines using a spring-loaded silo and delayed closure of the abdominal wall defect. In a metaanalysis, Kunz et al. reported that when the infants were randomly selected to a closure method, silo placement with delayed reduction was associated with reduced time to first feeding, ventilator days, and infection rates [29]. Fraga et al. indicated that primary closure, compared with silo placement, was associated with better outcomes, including the time to start enteral feeds, time to discontinue TPN, and hospital LOS [13]. However, Poola et al. reported that there were no significant differences between primary closure and delayed closure with silo in hospital LOS, time to enteral feeding, and ventilator days [30]. Our study also found similar results when we compared patients who experienced primary closure and staged closure with silo. We suggest that the main factor affecting the patients’ outcomes is whether the patient is a simple or complex GS rather than the abdominal wall closure method.

Mortality rates of GS and associated complications have decreased to <10% in most case series due to improvements in neonatal critical care and early and proper surgical management [31]. Our survival rate was comparable (93.2%) with high-income settings.

The main limitation of the study is the low sample size to determine and compare the adverse outcomes of patients, especially by gestational age at delivery. The strength of this study is that we performed this study at a single tertiary center, and all patients were managed with the consistent clinical and surgical treatment protocol.

## 5. Conclusion

This study indicated that simple GS, compared with complex GS, was associated with improved neonatal outcomes. We suggest that the main factor affecting the patients’ outcomes is whether the patient is a simple or complex GS rather than the abdominal wall closure method.

## Informed consent

The Ethics Committee of Kanuni Sultan Süleyman Training and Research Hospital approved the study (2020.07.160). We obtained informed consent forms from all parents.
